# Treatment with imatinib improves drug delivery and efficacy in NSCLC xenografts

**DOI:** 10.1038/sj.bjc.6603941

**Published:** 2007-08-21

**Authors:** G Vlahovic, A M Ponce, Z Rabbani, F K Salahuddin, L Zgonjanin, I Spasojevic, Z Vujaskovic, M W Dewhirst

**Affiliations:** 1 Department of Medicine – Oncology, Duke University Medical Center, PO Box 3335, Durham, NC 27710, USA; 2 Department of Biomedical Engineering, Duke University, Durham, NC, USA; 3 School of Medicine, Duke University, Durham, NC, USA; 4 Department of Radiation Oncology, Duke University Medical Center, Durham, NC, USA; 5 Department of Medicine, Durham Regional Hospital/Duke University Medical Center, Durham, NC, USA

**Keywords:** lung cancer, imatinib, drug delivery, interstitial fluid pressure

## Abstract

Imatinib, an inhibitor of PDGF-R*β* and other tyrosine kinase receptors, has been shown to decrease microvessel density and interstitial fluid pressure in solid tumours, thereby improving subsequent delivery of small molecules. The purpose of this study was to test whether pretreatment with imatinib increases the efficacy of traditional chemotherapy in mice bearing non-small cell lung carcinoma xenografts, and to investigate the effects of imatinib on liposomal drug delivery. Efficacy treatment groups included (*n*=9–10): saline control, imatinib alone (oral gavage, 100 mg kg^−1^ × 7 days), docetaxel alone (10 mg kg^−1^ i.p. 2 × /week until killing), and imatinib plus docetaxel (started on day 7 of imatinib). Tumours were monitored until they reached four times the initial treatment volume (4 × *V*) or 28 days. A separate experiment compared tumour doxorubicin concentrations (using high performance liquid chromatography) 24 h after treatment with liposomal doxorubicin alone (6 mg kg^−1^ i.v., *n*=9) or imatinib plus liposomal doxorubicin (*n*=16). Imatinib plus docetaxel resulted in significantly improved antitumour efficacy (0/10 animals reached 4 × *V* by 28 days) when compared to docetaxel alone (3/9 reached 4 × *V*, *P*=0.014) or imatinib alone (9/10 reached 4 × *V*, *P*=0.025). Pretreatment with imatinib also significantly increased tumour concentrations of liposomal doxorubicin. Overall, these preclinical studies emphasise the potential of imatinib as an adjunct to small molecule or liposomal chemotherapy.

Non-small cell lung carcinoma (NSCLC) has a limited response rate to current chemotherapeutics, with tumour shrinkage in only 20% of patients (by the RECIST criteria) and 2-year survival rates of 10–16% (Schiller *et al*, 2002). One of the major barriers to effective chemotherapy is the lack of adequate drug delivery to lung tumours, which has been linked to high tumour interstitial fluid pressures (IFPs) of up to 25 mm Hg (Jain, 1996). This challenge has also been demonstrated for many other solid tumours, including breast carcinoma, head and neck carcinoma, and melanoma (Jain, 1996; Heldin *et al*, 2004). Loss of pressure gradient across the microvessel wall inhibits drug transport from blood to the interstitium. In fact, elevated pressure throughout the core of solid tumours often results in drug distribution only to the periphery of tumours after intravenous injection (Boucher *et al*, 1990; Viglianti *et al*, 2004). Therefore, the rationale is quite strong for developing a method to improve tumour drug delivery by reducing IFP (Heldin *et al*, 2004; Jain, 2005).

Systemic chemotherapeutics encounter a number of obstacles that limit transport to solid tumours (Jain, 2001; Truskey *et al*, 2004). First, tumour angiogenesis results in immature vasculature with abnormal architecture and leaky, heterogeneous vessel walls (Yuan *et al*, 1995; Yuan, 1998). Vessels may also be compressed by the solid stress of proliferating tumour parenchyma and stroma (Boucher and Jain, 1992; Griffon-Etienne *et al*, 1999). These factors are known to impair vascular perfusion, thereby limiting drug access to the tumour. As noted above, another major problem is interstitial hypertension, which decreases convective transport of drug across the microvessel wall (Boucher *et al*, 1990; Heldin *et al*, 2004). Elevated IFP has been attributed to leakage of plasma proteins and fluid from permeable tumour vessels with high intravascular pressure. Tumours lack functional lymphatics to remove the excess interstitial fluid, and pressure equilibrates across the wall (Boucher and Jain, 1992). Furthermore, fibroblasts and pericytes have been shown to contribute to elevated IFP by exerting PDGF-mediated tension on the interstitial collagen matrix (Heldin *et al*, 2004). The PDGF receptors, located on stroma and perivascular cells, dimerise and bind PI3K, leading to integrin-mediated action on collagen fibers (Heuchel *et al*, 1999; Pietras *et al*, 2001).

Non-small cell lung carcinoma exhibits high IFP, upregulated PDGF, and a poor clinical prognosis, making this a particularly valuable model for impaired drug delivery (Kawai *et al*, 1997; Vlahovic *et al*, 2006). Interstitial hypertension particularly affects macromolecular therapies such as liposomes, antibodies, and proteins, as they require convective transport to cross the capillary wall (Jain and Baxter, 1988; Yuan, 1998; Netti *et al*, 1999). The current strategies for reducing IFP include blockage of cytokines (e.g. VEGF, PDGF, and TGF-*β*) and decompression of vessels by induction of tumour cell death (Boucher and Jain, 1992; Griffon-Etienne *et al*, 1999). Blocking VEGF prunes immature vessels, increases fraction of pericyte-covered vessels, and decreases vessel permeability, dilation and tortuosity (Tong *et al*, 2004; Willett *et al*, 2004; Jain, 2005). Inhibition of PDGF or PDGF-R*β* also prunes immature vessels, and additionally decreases contraction of the interstitial space and proliferation of stroma (Heuchel *et al*, 1999; Pietras *et al*, 2001; George, 2003; Matei *et al*, 2004; Vlahovic *et al*, 2006).

Imatinib (Gleevec, Novartis Pharmaceuticals Corp., East Hanover, NJ, USA) is a synthetic small molecule that inhibits phosphorylation of a number of tyrosine kinase receptors including PDGFR, c-kit, and bcr-abl (Buchdunger *et al*, 1995; George, 2003; Matei *et al*, 2004). It decreases proliferation of both tumour and stromal cells (George, 2003; Furuhashi *et al*, 2004; Kim *et al*, 2004; Matei *et al*, 2004; Ostman, 2004). In colonic xenografts, Pietras *et al* (2001) demonstrated that imatinib decreases IFP and thereby increases transcapillary transport of small molecules. We have recently reported the effects of imatinib on NSCLC xenografts, showing that 4 days of therapy (50 mg kg^−1^ gavage) significantly decreases IFP and microvascular density, lowers VEGF levels, and improves tumour oxygen delivery (Vlahovic *et al*, 2006). In the current study, we have continued this work by investigating whether pretreatment with imatinib can be used to affect tumour vasculature and consequently improve the efficacy of traditional chemotherapeutics in NSCLC xenografts. Furthermore, we provide a proof of principle for the use of imatinib to improve liposomal drug delivery in this model.

## MATERIALS AND METHODS

### Materials

Imatinib (Gleevec, STI571, Novartis Pharmaceuticals Corp.) was obtained from the hospital pharmacy in a 100-mg tablet form. Tablets were crushed and re-suspended in 0.9% NaCl. Liposomal doxorubicin (Doxil) was obtained from Ortho Biotech Products (Bridgewater, NJ, USA), and docetaxel (Taxotere) was obtained from Sanofi Aventis (Bridgewater). A human NSCLC line, A549, was purchased from American Type Culture Collection (Manassas, VA, USA).

### Non-small cell lung carcinoma xenograft model

Homozygous adult female athymic nude mice were housed in isolated caging with sterile rodent food and water *ad libitum*. Human NSCLC cells (A549) were grown in RPMI 1640 media supplemented with 5% bovine calf serum. The right hind limb of each mouse was inoculated subcutaneously with ∼5.0 × 10^5^ A549 cells, obtained from exponentially growing cell cultures, suspended in 200 *μ*l of HEPES-buffered saline. Xenograft tumour growth was monitored twice per week until tumours reached an average volume of 100 mm^3^. Xenograft volume (*V*) was determined by the following equation: *V*=(*L* × *W*^2^) × 0.5, where *L* is the length and *W* is the width of the flank tumour.

### Therapy and tumour harvest

After xenografts reached treatment volume (100 mm^3^), tumour-bearing animals were used in three separate studies. The first study compared vascular maturity (by immunohistochemistry) after treatment with imatinib (*n*=4) *vs* control (saline, *n*=5). Imatinib was administered via gavage once a day at a dose of 100 mg kg^−1^ for 7 days, and saline was administered following the same schedule. Animals were killed 24 h after the last imatinib injection, and tumours were harvested and snap frozen at −80°C.

The second study compared tumour growth times after treatment with control (saline plus saline), imatinib alone (plus saline), docetaxel alone (plus saline), or imatinib plus docetaxel (*n*=9–10 mice per group). Docetaxel was administered intraperitoneally at a dose of 10 mg kg^−1^ twice per week until killing, and saline was administered following the same schedule. Injections of docetaxel (docetaxel±imatinib groups) or intraperitoneal saline (control+imatinib alone groups) were initiated on day 7 of imatinib or saline pretreatment. After therapy, animals were monitored as they recovered from anaesthesia. Tumour volumes were measured three times per week for 28 days or until the tumour volume reached four times the volume on the first day of treatment.

The third study compared tumour doxorubicin concentrations after treatment with liposomal doxorubicin alone (*n*=9) *vs* imatinib plus liposomal doxorubicin (*n*=16, randomised in 1 : 2 ratio). Liposomal doxorubicin was delivered intravenously as a single dose (6 mg kg^−1^). When combined with imatinib, liposomal doxorubicin was delivered on day 7 of imatinib treatment. Animals were killed 1 day (24 h) after liposomal doxorubicin injection, and tumours were harvested and snap frozen at −80°C. All animal protocols were approved by the Duke University Animal Care and Use Committee. Experiments were performed in accordance with the Interdisciplinary Principles and Guidelines for the Use of Animals in Research, Marketing, and Education (New York Academy of Sciences, New York, NY, USA), as well as the UKCCCR guidelines (Anon, 1988).

### Immunohistochemical staining

Vascular markers for this study included the endothelial cell markers CD31 and CD105, as well as smooth muscle alpha-actin (*α*-SMA). CD31 was used to stain all endothelial cells, whereas CD105 was used to indicate immature endothelial cells. Smooth muscle *α*-actin was used to stain pericytes covering microvessels. Frozen tumours harvested from control and imatinib alone groups were cut into 10–12 *μ*m sections using a LEICA CM 1850 cryotome (Meyer Instruments Inc., Houston, TX, USA). Consecutive tissue sections were cut at the largest circumference of the tumour and placed onto poly-L-lysine-coated slides (Polysciences Inc., Warrington, PA, USA). Cryosections were then split into two sets for staining. All antibodies were diluted in phosphate-buffered saline (PBS) with 0.1% bovine serum albumin and 0.1% Tween-20 (PBS-BT).

In the first set, sections were stained for CD31 and *α*-SMA. Cryosections were air dried and fixed for 10 min in acetone at 4°C. Tumour sections were then blocked with 10% normal donkey serum for 15 min and rinsed three times for 2 min in PBS. Sections were probed sequentially with primary antibodies against CD-31 (rat anti-mouse, diluted 1 : 100, BD Pharmingen, BD Biosciences, San Jose, CA, USA) and *α*-SMA (mouse monoclonal antibody, clone IA4, diluted 1 : 400, Lab Vision Corp., Fremont, CA, USA). After rinsing again with PBS, primary antibodies were respectively revealed with secondary antibodies coupled to TRITC (red, for CD-31) or FITC (green, for *α*-SMA) (Jackson Immunoresearch, West Grove, PA, USA).

In the second set, sections were stained for CD31 and CD105. Sections were fixed for 1 h in 4% paraformaldehyde in PBS (pH 7.2). Endogenous peroxidase activity was quenched with 3% hydrogen peroxide for 15 min, and tumour sections were then blocked with 10% normal donkey serum for 15 min and rinsed three times for 2 min in PBS. Consecutive sections were incubated overnight at 4°C with rat monoclonal antibody to CD31 diluted 1 : 200 or CD105 diluted 1 : 400 (BD Biosciences, San Jose, CA, USA). After rinsing again with PBS, primary antibodies were enhanced with biotinylated donkey anti-rat secondary antibody (diluted 1 : 100) for 30 min at room temperature (Jackson Immunoresearch). Finally, an avidin-biotin complex was applied (Vectastain ABC kit, Vector Lab Inc., Burlingame, CA, USA). Markers were visualised with 3,3′-diaminobenzidine tetrahydrochloride (DAB chromogen, Vector Lab Inc.).

### Image analysis

Tumour sections were quantitatively analysed using a semiautomatic computerised digital image analysis system (Metamorph Imaging System, Molecular Devices Corp, Sunnyvale CA, USA). A high-resolution intensified solid-state camera was mounted on a fluorescence microscope (Axioskop, Zeiss, Axioskop Zplus, Carl Zeiss Inc., Germany) with a computer-controlled motorised stepping stage. For the first set (CD31 *vs α*-SMA), digitised images were acquired with both TRITC and FITC filters, and images were overlaid using the Metamorph software. Microvessel density was determined by counting CD31-positive structures in five random fields per tumour, and *α*-SMA-positive vessels were counted from among these structures.

For the second set (CD31 *vs* CD105), quantification of vessels was performed using the Image J ‘Analyze Particles’ function by a blind observer without any manipulation in brightness or contrast. The threshold intensity was manually set above the background staining intensity, and the Image J function selected for stained vessels with a minimum size of 10 pixels per vessel. The average number of CD31-stained vessels per region of interest (over three regions per animal) was calculated as the microvessel density. The average number of CD105-stained vessels in the same regions was calculated from the consecutive section.

### High performance liquid chromatography for doxorubicin concentration

Tumours harvested from the liposomal doxorubicin group and the imatinib plus liposomal doxorubicin group were cryo-crushed at −80°C, thawed, diluted, and homogenised. Three samples were analysed from each tumour. Doxorubicin was extracted from the homogenate using chloroform and silver nitrate. The organic phase was separated, dried, and reconstituted in isopropanol. Doxorubicin concentration was then measured by high performance liquid chromatography (HPLC) with fluorimetry (Cummings, 1985; Kong *et al*, 2000). A standard concentration set was prepared using tumour homogenate and known concentrations of doxorubicin. To correct error in weight measurement due to variable tissue water content, concentrations were normalised by total water-soluble protein concentration, which was measured by the Bradford assay (colorimetric response of Coomassie Blue G dye).

### Statistical methods

Descriptive summary statistics are presented as mean±s.e. of the mean, except for the time-to-four-times-tumour-volume data, which are expressed as median values with 95% confidence intervals. Statistical analyses of doxorubicin concentration and histologic data were performed using the two-sample Student's *t*-test. For the antitumour efficacy data, the primary end point (time to reach four times the original tumour volume) was analysed using the Cox proportional hazards model to compare groups. Censoring was taken into account for animals that did not reach four times the original treatment volume by the time they were killed (at 28 days). *P*-values less than 0.05 were considered to be statistically significant.

## RESULTS

Since imatinib is known to inhibit PDGF-R*β* and decrease IFP in solid tumours, it has the potential to ‘normalise’ the tumour microenvironment and thereby improve delivery and efficacy of chemotherapeutic agents. With this rationale, we performed three *in vivo* translational studies on the effects of imatinib in NSCLC xenografts. We first used histology to test the vascular effects of imatinib, specifically assessing microvessel density (CD31 positivity), pericyte coverage (*α*-SMA positivity), and endothelial cell immaturity (CD105 positivity). Then, we followed up on recently published data that demonstrate that pretreatment with imatinib increases delivery of small molecules to tumours (Pietras *et al*, 2001; Vlahovic *et al*, 2006). We evaluated the antitumour efficacy of imatinib plus docetaxel (a small molecule chemotherapeutic commonly used for NSCLC treatment) in comparison with either agent alone. Finally, as elevated IFP is known to be a major barrier to convective transport of liposomes, we tested whether pretreatment with imatinib can be used to increase tumour delivery of liposomal doxorubicin. These three studies were designed to complement previously published data and increase the understanding of PDGFR*β*-mediated effects of imatinib in NSCLC.

**Figure 1:**
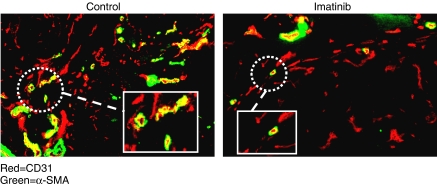
Representative fluorescent images of NSCLC xenograft sections after treatment with saline control or imatinib, 100 mg kg^−1^ × 7 days. Images show endothelial cells stained with CD31 (red) and pericytes stained with anti-*α*-SMA (green).

**Figure 2:**
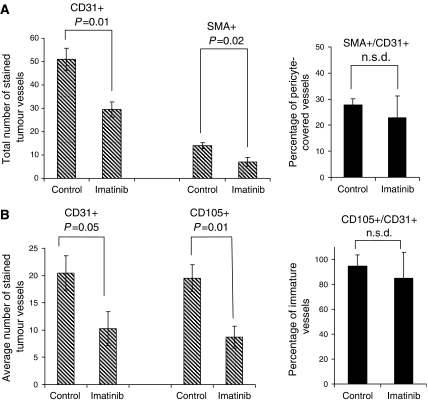
Vascular markers in NSCLC xenografts after treatment with saline control or imatinib, 100 mg kg^−1^ × 7 days. (**A**) The total numbers of anti-CD31- and anti-*α*-SMA-stained vessels were measured in five regions of the same tumour section. The number of anti-CD31-stained vessels represents total microvessel density, and the number of anti-*α*-SMA-stained vessels indicates the extent of pericyte coverage. Data represent mean±s.e. (**B**) The average numbers of anti-CD31- and anti-CD105-stained vessels were measured in three regions of consecutive tumour sections. The number of anti-CD105-stained vessels indicates relative endothelial cell immaturity. The number of anti-CD31-stained vessels differs from (**A**) due to distinct methods.

Representative images of NSCLC microvessels stained for CD31 (red) and *α*-SMA (green) are shown in Figure 1 after treatment with saline (control) or imatinib. Pericytes (green) can be seen overlying the endothelial cells (red), especially in larger vessels. After a 7-day course of imatinib treatment, the total number of vessels (anti-CD31-stained) decreased significantly from 51.0±4.7 to 29.5±3.2 (s.e., *P*=0.01, Figure 2A). The number of anti-*α*-SMA-stained microvessels, representing mature pericyte-covered vessels, also decreased significantly, from 14.1±1.2 to 7.1±1.3 (*P*=0.02). The percentage of total vessels that were positive for *α*-SMA lowered from 28 to 23% (Figure 2A), indicating a trend towards decreased pericyte coverage after treatment with imatinib (not significantly different).

In a separate set of tumour sections, the average numbers of CD31-stained and CD105-stained vessels were compared in consecutive sections (Figure 2B). Owing to different image analysis methods, the absolute number of CD31-stained vessels in this experiment (Figure 2B) was lower than in the former experiment (Figure 2A), but treatment with imatinib again resulted in a significant decrease in vessel density (*P*=0.05). The density of immature CD105-stained endothelial cells in the tumour decreased from 19.5±2.5 to 8.7±2.0 after treatment with imatinib (*P*=0.01), and the ratio of CD105- to CD31-stained (total) vessels was used as an indicator of vascular immaturity. This ratio was 0.95±0.09 in the control tumour and 0.85±0.21 after imatinib therapy (Figure 2B), showing a trend towards decreasing vessel immaturity (or increasing maturity).

**Figure 3:**
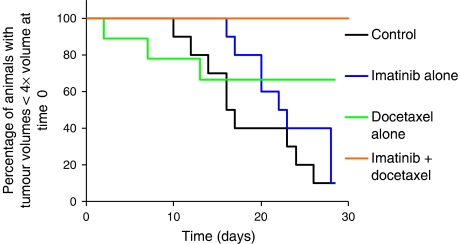
Antitumour response in NSCLC xenografts after treatment with saline, imatinib alone, docetaxel alone, or imatinib plus docetaxel (*n*=9–10 mice per group). Imatinib dose was 100 mg kg^−1^ oral gavage × 7 days. Docetaxel dose was 10 mg kg^−1^ i.p. twice per week, starting on day 7 of imatinib or saline treatment. Tumours were monitored three times per week until they reached four times the initial treatment volume (4 × *V*) or 28 days. The Kaplan–Meier plot represents the percentage of animals with tumour volumes less than four times the volume at the beginning of treatment, as a function of time after treatment.

Figure 3 clearly shows that the combination of imatinib plus docetaxel was significantly more effective than either agent alone in the NSCLC xenograft. When docetaxel was administered after 7 days of imatinib treatment, tumour growth was delayed such that no animals reached the end point of four times the original tumour volume (4 × *V*) by 28 days after therapy. In comparison, three out of nine animals receiving docetaxel alone reached 4 × *V* by 28 days, showing a significantly higher likelihood ratio for reaching 4 × *V* (*P*=0.014 *vs* combination). Almost all (9 out of 10) animals receiving imatinib alone reached this end point, with a median tumour growth time of 22.5±3.0 days (95% CI). Again, imatinib alone showed a significantly higher likelihood ratio for reaching 4 × *V* (*P*=0.025 *vs* combination).

**Figure 4:**
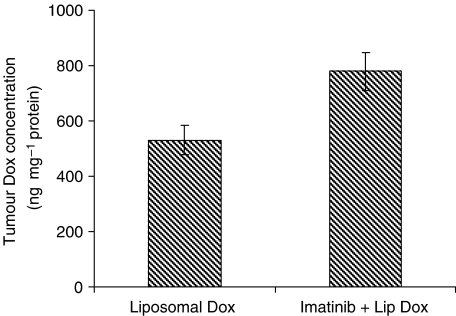
Tumour doxorubicin concentrations (ng doxorubicin per mg protein) 24 h after therapy with liposomal doxorubicin alone or imatinib plus liposomal doxorubicin. Liposomal doxorubicin was administered i.v. at a dose of 6 mg kg^−1^. For combined therapy, liposomes were administered on day 7 of imatinib treatment (100 mg kg^−1^ oral gavage × 7 days). Tumour doxorubicin concentration was measured by HPLC, and data represent mean±s.e.

The assessment of imatinib plus liposomal doxorubicin showed that prior treatment with imatinib improves delivery of liposomal doxorubicin to NSCLC tumours (Figure 4). A single dose of 6 mg kg^−1^ liposomal doxorubicin (without imatinib) resulted in a mean tumour doxorubicin concentration of 530.4±53.5 ng doxorubicin per mg protein at 24 h after injection (*n*=9). Pretreatment with 7 days of 100 mg kg^−1^ imatinib gavage significantly increased the tumour doxorubicin concentration to 779.2±68.8 ng doxorubicin per mg protein (*n*=16, *P*=0.02).

## DISCUSSION

Imatinib has been implicated as a possible effector of vascular ‘normalisation’, the therapeutic paradigm proposed by Jain (2005) to improve drug and oxygen delivery to tumours. Indeed, in preclinical studies, imatinib therapy has normalised the tumour microenvironment by decreasing IFP, increasing transcapillary transport of oxygen and ^51^Cr-EDTA, and lowering VEGF and microvessel density (Pietras *et al*, 2001; Vlahovic *et al*, 2006). The current study further demonstrates a significant decrease in density of immature endothelial cells (CD105+), with a trend towards higher endothelial cell maturity (lower CD105–CD31 ratio, Figure 2B). In addition, Figure 2A supports a trend towards decreased fraction of pericyte coverage after imatinib therapy. Although not a ‘normalising’ effect, this result is consistent with imatinib inhibiting the actions of PDGF and PDGF-R*β* in pericyte recruitment and vessel wall stabilisation (George and Kaelin, 2003; Ostman, 2004). Importantly, dosing and scheduling may be crucial if ‘normalisation’ is the goal (Winkler *et al*, 2004; Jain, 2005). Future studies should determine the temporal windows of IFP reduction and vascular remodelling induced by imatinib in order to optimise combined therapy schedules (Jain, 2005).

This is the first study to demonstrate the *in vivo* efficacy of imatinib, as an adjunct to chemotherapy in NSCLC treatment. For NSCLC, the most commonly used first line chemotherapeutics include platinum, taxanes, gemcitabine, and vinorelbine (Schiller *et al*, 2002). The taxane docetaxel was chosen for this preclinical study, because it is FDA approved as a second line single-agent therapy, and it is under clinical investigation as a first-line single-agent therapy. Here we show that imatinib plus docetaxel resulted in significantly improved antitumour efficacy when compared to docetaxel alone or imatinib alone. These results, along with previously published studies (Pietras *et al*, 2001; Vlahovic *et al*, 2006), suggest that imatinib improves small molecule drug delivery, which is typically thought to be governed by diffusion (Heldin *et al*, 2004). This may be linked to an increase in diffusion by an unknown mechanism, possibly related to vessel remodelling or available volume fraction in the extracellular matrix (Heuchel *et al*, 1999; Ostman, 2004). However, some evidence suggests that the effects of convection on small molecules may be underestimated, especially in the tumour microenvironment (Pietras *et al*, 2001; Heldin *et al*, 2004; Ostman, 2004). Further preclinical studies should investigate the drug delivery effects of this type of combination therapy, and expand application to other tumour models and other chemotherapeutic agents.

This study also provides a proof of principle for the enhancement of liposomal (i.e. macromolecular) drug delivery by reduction of IFP (Yuan *et al*, 1994, 1995). Macromolecular transport is dominated by convection, which is governed by hydrostatic and oncotic pressures (Starling forces) (Truskey *et al*, 2004). A decrease in interstitial hydrostatic pressure is expected to increase the transport of macromolecules across the vessel wall (Heldin *et al*, 2004). Thus, the observed increase in liposomal doxorubicin delivery to NSCLC after pretreatment with imatinib can be explained by the decrease in IFP previously reported by this group in NSCLC (Figure 4, Pietras *et al*, 2001; Vlahovic *et al*, 2006).

Overall, the observed effects of imatinib in NSCLC are consistent with the inhibition of PDGFR-*β*; these changes in the microenvironment, including decreased IFP and modified vasculature, result in improved drug delivery and efficacy in the preclinical setting. Currently, this hypothesis is being further tested in clinical trials in which patients with metastatic NSCLC are treated with standard cisplatin and docetaxel plus concurrent imatinib. All enrolled patients must be PDGFR-*β* positive by immunohistochemistry, and treatment response is monitored by the RECIST criteria. Patients also participate in a lead-in portion of the study in which dynamic contrast-enhanced (DCE) MRI is performed before and after a 7-day course of imatinib alone, to explore DCE-MRI as a biomarker for changes in the tumour microenvironment. Preliminary data have revealed that DCE-MRI is feasible and demonstrates a decrease in tumour leakage space (interstitial or extracellular extravascular space) after treatment with imatinib. This suggests a decrease in IFP that may imply improvement in tumour drug delivery. However, a major challenge to clinical research in drug delivery is the lack of non-invasive drug imaging methods (Viglianti *et al*, 2004; Ponce *et al*, 2007). The phase I portion of the clinical trial is completed, and the phase II portion evaluating the efficacy of combined imatinib plus chemotherapy is currently successfully enrolling. In conclusion, our preclinical studies with imatinib in NSCLC demonstrate its potential to improve tumour drug delivery and efficacy, and this strategy was subsequently rapidly translated into clinical trials.
